# Non-squamous cell carcinoma diseases of the larynx: clinical and imaging findings^☆^^☆☆^

**DOI:** 10.1016/j.bjorl.2019.02.003

**Published:** 2019-03-16

**Authors:** Serap Doğan, Alperen Vural, Güven Kahriman, Hakan İmamoğlu, Ümmühan Abdülrezzak, Mustafa Öztürk

**Affiliations:** a Erciyes University Medical Faculty, Department of Radiology, Kayseri, Turkey; b Erciyes University Medical Faculty, Department of Otorhinolaryngology, Kayseri, Turkey; c Erciyes University Medical Faculty, Department of Nuclear Medicine, Kayseri, Turkey

**Keywords:** Larynx, non-squamous cell neoplasms, Laryngeal neoplasm, Inflammatory laryngeal lesions, Laringe, neoplasias de células não escamosas, Neoplasia laríngea, Lesões laríngeas inflamatórias

## Abstract

**Introduction:**

Squamous cell carcinoma is the most common laryngeal neoplasm and accounts for approximately 95% of all malignant neoplams of the larynx. However, various benign and malignant tumors and inflammatory diseases may affect the larynx.

**Objective:**

The purpose of this study is to analyze the clinical and imaging findings of non-squamous cell neoplasms and inflammatory diseases of the larynx.

**Methods:**

This retrospective study was conducted in 18 patients who were diagnosed with non-squamous cell carcinoma lesions of larynx at our institution between 2007-2017. Clinical symptoms, examination findings, imaging characteristics, histopathologic diagnosis and treatment modalities were analyzed.

**Results:**

There were 9 malignant lesions (2 chondrosarcoma, 1 neuroendocrine tumor-atipical carcinoid, 1 Natural Killer/T-cell lymphoma, 1 diffuse large B-cell lymphoma, 3 plasmocytoma-multiple myeloma involvement, 1 adenocarcinoma metastasis), 3 benign neoplasms (chondroma, paraganglioma, lipoma), 2 tumor-like lesions (Brown tumor and inflammatory myofibroblastic tumor), 3 inflammatory lesions (Wegener granulomatosis, Behçet's disease and tuberculosis involvements), and 1 vascular malformation. The most common presenting symptom was hoarseness (66.6%). Paraganglioma was seen as hypervascular lesion on computed tomography and magnetic resonance imaging and showed intense tracer uptake on 68Gallium-DOTA-peptide PET/CT. Chondroid matrix calcifications were detected in chondroma and chondrosarcoma-grade 1. In patients with vascular malformation and lipoma, the typical imaging findings made it possible to diagnose.

**Conclusion:**

Imaging studies may provide clues for diagnosis of non-squamous cell laryngeal lesions. Clinical and imaging findings and previous clinical history should be evaluated together in clinical management of laryngeal lesions.

## Resumo

**Introdução:**

O carcinoma de células escamosas é a neoplasia laríngea mais comum, representa aproximadamente 95% de todas as neoplasias malignas da laringe. No entanto, vários outros tumores benignos e malignos, e doenças inflamatórias, podem afetar a laringe.

**Objetivo:**

O objetivo deste estudo é analisar os achados clínicos e de imagem de neoplasias de células não-escamosas e de doenças inflamatórias da laringe.

**Método:**

Este estudo retrospectivo foi feito com 18 pacientes diagnosticados com lesões de carcinoma de células não escamosas da laringe em nossa instituição, entre 2007–2017. Foram analisados os sintomas clínicos, achados dos exames, características de imagens, diagnóstico histopatológico e modalidades de tratamento.

**Resultados:**

Foram identificados 9 casos com lesão maligna (2 condrossarcomas, 1 tumor carcinoide neuroendócrino atípico, 1 linfoma de células T/NK, 1 linfoma difuso de grandes células B, 3 plasmocitomas com envolvimento de mieloma múltiplo, 1 metástase de adenocarcinoma, 3 neoplasias benignas (condroma, paraganglioma, lipoma), 2 lesões “tumor like” (tumor de Brown e tumor miofibroblástico inflamatório), 3 lesões inflamatórias (granulomatose de Wegener, doença de Behçet e tuberculose) e 1 malformação vascular. O sintoma mais comum foi a rouquidão (66,6%). O paraganglioma foi visto como lesão hipervascular na tomografia computadorizada e na ressonância magnética, e mostrou intensa captação do traçador na PET/TC com 68Gálio-DOTA. Calcificações de matriz condroide foram detectadas no condroma e condrossarcoma grau 1. Em pacientes com malformação vascular e lipoma, os achados típicos de imagem tornaram possível o diagnóstico.

**Conclusão:**

Estudos de imagem podem fornecer pistas para o diagnóstico de lesões laríngeas de células não escamosas. Achados clínicos e de imagem e histórico clínico prévio devem ser avaliados em conjunto no manejo clínico das lesões laríngeas.

## Introduction

Squamous Cell Carcinoma (SCC) is the most common laryngeal neoplasm and constitutes approximately 95% of all malignant neoplams of the larynx. However, diverse benign and malignant tumors and a variety of inflammatory diseases may affect the larynx. True benign tumors account for 5% or less of all the laryngeal tumors.^1^ The routine clinical approach to laryngeal lesions include indirect laryngoscopy and subsequent biopsy at direct laryngoscopy. Squamous cell carcinomas usually manifest as mucosal lesions and are easily visible at laryngoscopy, whereas non-SCC laryngeal tumors generally present as submucosal masses. Although endoscopy is the gold standard technique for the evaluation a mucosal lesion, it may not a reliable method for the assessment of submucosal structures and any possible deep extension of the lesion.

In the diagnostic work-up of laryngeal lesions, Multidetector Computed Tomography (MDCT) is the first choice cross-sectional imaging modality. Thin slices MDCT provides high spacial resolution and allows high quality multiplanar reformation. Exact location, vascularization and extension of lesions, involvement of laryngeal skeleton can all be evaluated with soft tissue and bone window settings. MDCT plays also a crucial role for determining the appropriate biopsy site, especially in submucosal lesions with overlying normal mucosa.

Magnetic Resonance Imaging (MRI) has the potential for better tissue characterization because of high soft tissue contrast resolution. MRI provides a more accurate and detailed information especially in evaluation of submucosal spaces, anterior commissure, subglottis, cartilage infiltration and tongue base infiltration.^2^ Reported sensitivity values of MRI in the assessment of preepiglottic space, paraglottic space and cartilage invasions are 91%–100%, 93%–97% and 89%–94%, respectively.^2^, ^3^, ^4^ Both MDCT and MRI also provide the assessment of cervical lymph node involvement.

There are very few studies in the English literature investigating uncommon laryngeal diseases and most of them are case reports. In this study, we aimed to analyze clinical and imaging findings of non-SCC laryngeal neoplasms and inflammatory diseases as well as highlighting the distinguishing features that are useful for diagnosis and clinical management of these rare diseases.

## Methods

This single institutional study was approved by ethics committee of our university (decision number: 2017/43). Informed consent was waived due to retrospective design of the study. We retrospectively analyzed the data of 18 patients (6 females, 12 males) who were diagnosed and treated with non-SCC laryngeal neoplasms and inflammatory diseases at radiology, nuclear medicine and head and neck surgery departments of our university from 2007 to 2017. Clinical charts, imaging and videolaryngoscopic findings, and pathological reports were reviewed to determine patients’ age and gender, clinical symptoms, examination findings, imaging characteristics, histopathologic diagnosis and treatment modalities. Multidetector Computed Tomography (MDCT) images of 16 patients, MR images of 6 patients, Fluorine-18 (F-18) Fluorodeoxyglucose (FDG) Positron Emission Tomography/Computed Tomography (PET/CT) images of 4 patients and Gallium-68 (68Ga) DOTA-peptide PET/CT images of 1 patient were available in picture archiving and communication system. Three patients (diagnosed with granulation tissue and radio-necrosis) who were treated with surgery, chemotherapy or radiation therapy before imaging were excluded from the study.

## Results

The mean age of all patients at presentation was 55.2 years (range, 23–85 years). The mean age of patients who were diagnosed with benign lesions and malignant lesions was 50.5 and 60 years, respectively.

Histopathologic diagnosis of laryngeal lesions in 14 patients were obtained by endoscopic laryngeal biopsy or total mass excision. The remaining 4 patients were diagnosed with previous clinical history, imaging findings, laboratory findings and follow-up. Of these, in one patient with primary hyperparathyroidism, parathyroid adenoma and multiple lytic expansile bone lesions in the maxilla and mandible were seen on CT images. The laryngeal lesion of this patient was located within the cricoid cartilage. Previous mandible biopsy result (osteoclast type multinucleated giant cells in proliferative fibrous tissue) was compatible with brown tumor of hyperparathyroidism. The laryngeal lesion of this patient was diagnosed as a Brown tumor. In a patient with Wegener's Granulomatosis (WG), previous lung biopsy result was small vessel vasculitis. Laboratory findings and lung CT findings were compatible with WG. Increased soft tissue thickness and diffuse laryngeal edema were seen on neck CT and these findings resolved after treatment. Vascular malformation was diagnosed based on the imaging findings. The last patient had previously diagnosed multiple myeloma. Laryngeal lesion size decreased after multiple myeloma treatment.

**Figure 1:**
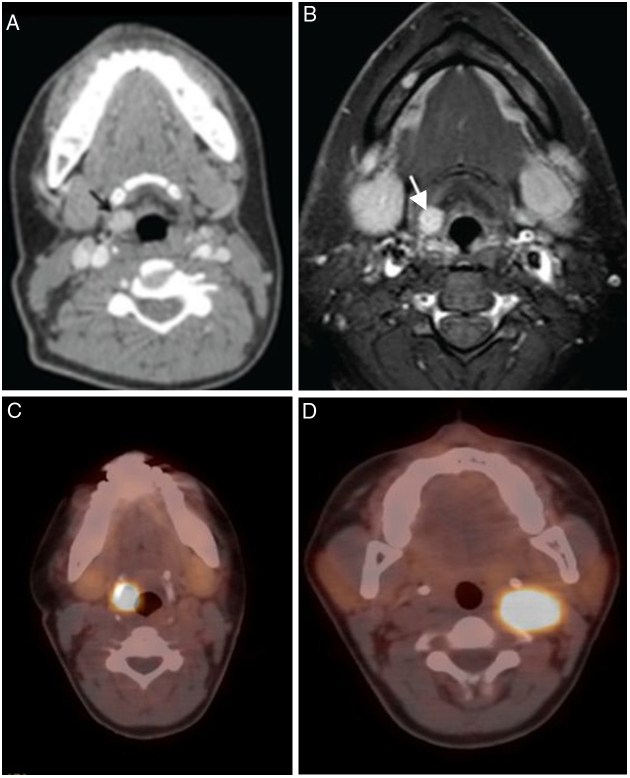
(A–D) 34 year-old woman with laryngeal paraganglioma. Right preepiglottic well defined enhanced mass (arrows) is seen in contrast enhanced CT (A) and contrast enhanced T1 weighted turbo spin echo spectral fat saturation inversion recovery (T1 TSE SPIR); (B) images. Axial 68Gallium-DOTA-peptide PET/CT fusion image (C); shows intense uptake by the right laryngeal paraganglioma similar with syncronous left carotid body paraganglioma (D) (figures of this case were printed in Ref. 5).

The most common clinical presentations were hoarseness (12/18, 66.6%), dyspnea (6/18, 33.3%) and dysphagia (4/18, 22.2%). There was only one asymptomatic laryngeal lesion which was diagnosed with paraganglioma. Laryngeal paraganglioma was detected incidentally on neck CT during the evaluation of synchronous left carotid body paraganglioma (Fig. 1).^5^ This patient presented with a left- sided mass in the neck. Physical examination findings and treatment modalities of all patients were summarized in Table 1.

**Table 1:** Clinicopathological data of patients.

	Age (years)/Gender	Clinical presentation	Physical examination findings	Diagnosis	Approach and treatment
1	85/F	Hoarseness, stridor, dyspnea	Large subglottic mass.	Chondrosarcoma (grade 1)	Total laryngectomy
2	82/M	Hoarseness	Left sided transglottic mass	Chondrosarcoma (grade 2)	Larynx biopsy
					Total laryngectomy + left neck
					Dissection
3	68/M	Hoarseness	Mass involving right aryepiglottic fold and interarytenoid mucosa.	Neuroendocrine tumor Grade 2 (atipical carcinoid tumor)	Larynx biopsy
					Chemoradiotherapy
4	39/F	Hoarseness, dysphagia, right ear pain	Mass in the laryngeal side of epiglottis	Extranodal natural killer/T cell lymphoma	Larynx biopsy
					Chemoradiotherapy
5	23/F	Hoarseness	Yellowish mass involving the left aryepiglottic fold, band ventricle and tongue base	Posttransplant lymphoproliferative disease, monomorphic, diffuse large B-cell type	Larynx and lung bx
					Systemic treatment for lymphoma
6	44/M	Dyspnea	Subglottic mass narrowing the passage.	Plasmocytoma-multiple myeloma involvement	Larynx biopsy
					Systemic treatment for multiple myeloma
7	55/M	Dyspnea, hoarseness	Transglottic mass	Plasmocytoma-multiple myeloma involvement	Spinal biopsy Systemic treatment for multiple myeloma
8	70/M	Dyspnea, dysphagia	Mass in the postcricoid region	Plasmocytoma-multiple myeloma involvement	Larynx biopsy
					Systemic treatment for multiple myeloma
9	74/M	Hoarseness	Right sided glottic mass	Adenocarcinoma metastasis (histopathologic features were similar with lung adenocarcinoma, operated 2 years a go).	Larynx biopsy
10	34/F	Left sided neck mass (laryngeal lesion was asymptomatic)	Normal laryngoscopic findings and palpable mobile, 1.5 cm mass anterior to the sternocleidomastoid muscle on left side.	Paraganglioma	Excision of laryngeal and carotid body paragangliomas after angiographic embolization of vascular supplies on two separate operations.
11	70/M	Hoarseness, dysphagia	Immobile left arytenoid, glottic space obstructed.	Chondroma	Urgent tracheotomy, laryngofissure surgery
12	68/F	Hoarseness	Right sided soft submucosal supraglottic and glottic mass.	Laryngeal lipoma	Excision of laryngeal lipoma
13	63/M	Hoarseness	Left sided mass involving the left vocal cord and anterior commissure.	Inflammatory myofibroblastic tumor-left vocal cord squamous cell carcinoma-anterior commissure.	Larynx biopsy
					Laser cordectomy + postoperative radiotherapy
14	52/F	Dyspnea, a mass in the jaw	Subglottic mass narrowing the passage	Brown tumor of hyperparathyroidism Parathyroid adenoma	Parathyroidectomy
15	30/M	Dyspnea, stridor	Bilateral limited vocal cord mobility, deformity of epiglottis.	Wegener's granulomatosis involvement.	Immune supressive therapy
16	61/M	Hoarseness	Left ventricle and vocal cord edema.	Granulomatous inflammation	Larynx biopsy
			Left supraglottic mass lesion	Tuberculous involvement	Antituberculous medical treatment.
					Lung biopsy (Caseified granulomatous inflammation).
17	42/M	Dysphagia	An ulcerated lesion on the left arytenoid,	Chronic-active inflammation Behçet's disease involvement	Larynx biopsy
					Immune supressive therapy
18	35/M	Hoarseness and right sided neck mass	Purple colored transglottic mass right side of the larynx	Vasculary malformation (low-flow)	The patient cannot follow-up

**Figure 2:**
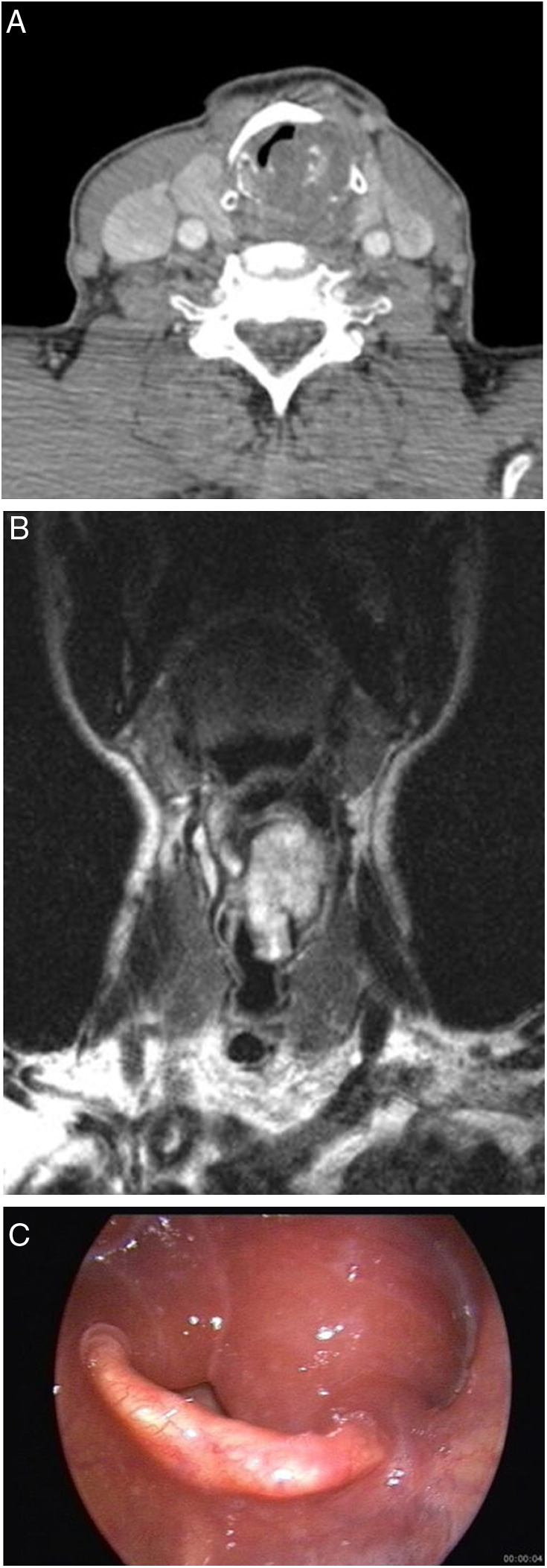
(A–C) 70 year-old man with laryngeal chondroma. Contrast enhanced CT image (A) shows expansile mass arising from cricoid cartilage. Chondroid calcifications are seen within the mass. Coronal T2 weighted TSE image (B) shows high signal intensity transglottic mass. Endoscopy (C) reveals bilateral edema of the mucosa of the arytenoid cartilages and a large submucosal mass.

**Figure 3:**
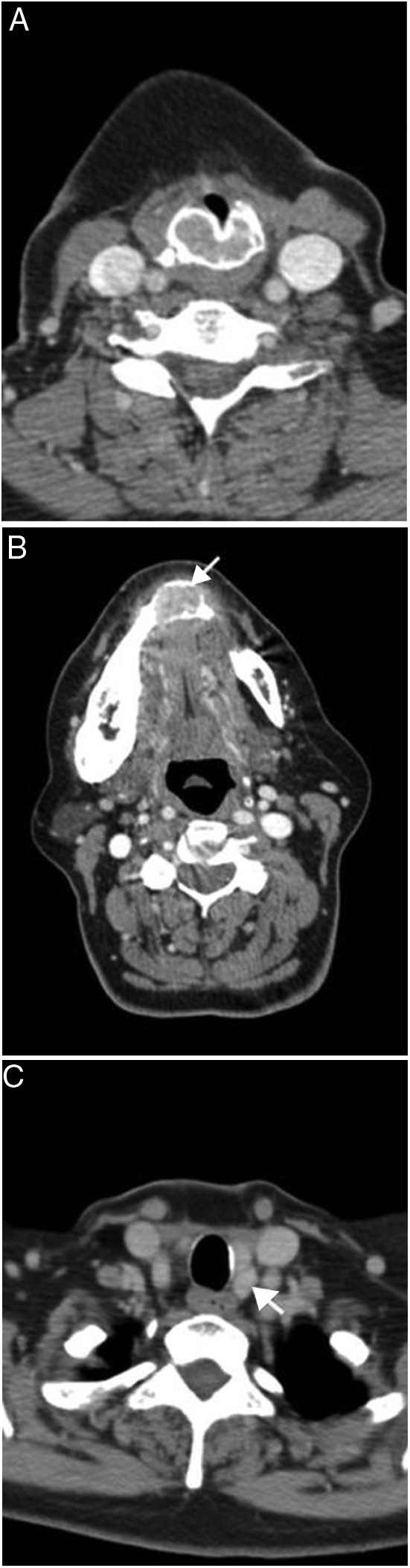
(A–C) 52 year old woman with primary hyperparathyroidism and multiple brown tumors. Contrast enhanced CT image (A) shows expansile mass-brown tumor arising from the cricoid cartilage. Mandibular lytic expansile mass is seen on more superior level CT image (B). Parathyroid adenoma is seen at left inferior parathyroid gland location (C).

The mean size of malignant and benign lesions was 28 mm and 23.6 mm, respectively. Lesion size was not measured in the patient with WG due to diffuse soft tissue involvement (edema and soft tissue thickening) of lesion. Locations of lesions are presented in Table 2. The origin of the tumor was cricoid cartilage in patients with chondroma, chondrosarcoma-Grade1, Brown tumor and in 2 of 3 multiple myeloma involvements (Figure 2, Figure 3).

**Table 2:** Diagnostic imaging characteristics of patients.

Patient no.	Diagnosis	Maximum lesion size (mm)	Lesion location	CT	MRI	Additional imaging modalities
1	Chondrosarcoma (Grade 1)	30	Subglottic	Heterogeneous expansile mass arising from cricoid cartilage with internal chondroid matrix calcification	T1W isointense, T2W hyperintense mass, minimally heterogeneous enhancement	–
2	Chondrosarcoma (Grade 2)	60	Supraglottic + glottic + subglottic	Transglottic hypodense mass with central necrosis.	–	–
				Extralaryngeal extension.		
				Thyroid and cricoid cartilage destruction.		
				Level 6 metastatic lymphadenopathy.		
3	Neuroendocrine tumor Grade 2 (atipical carcinoid tumor)	16	Supraglottic	Mass on right aryepiglottic fold and interarytenoid region	T1W isointense, T2W hyperintense, enhancing soft tissue	–
4	Extranodal Natural killer/T cell lymphoma	30	Supraglottic + glottic	Mass on the epiglottis, aryepiglottic fold, band ventricle, vocal Cord.	–	F-18 FDG PET/CT
				Ipsilateral level 2 metastatic lymphadenopathy.		Increased tracer uptake in right supraglottic soft tissue (SUVmax 2.1).
						Increased tracer uptake in ipsilateral level 2 lymph nodes (SUVmax 2.6)
5	Posttransplant lymphoproliferative disease, monomorphic, diffuse large B-cell type	16	Supraglottic	Mass on left aryepiglottic fold, band ventricle,	–	F-18 FDG PET/CT
				Left level 3 metastatic lymphadenopathy		Intense tracer uptake in oropharynx and supraglottic larynx (SUVmax 11.5),
						Increased tracer uptakes in lung, pleura, spleen, breast, stomach, axillary, hilar and cervical lymph nodes.
6	Plasmocytoma-multiple myeloma involvement	28	Subglottic	Lytic-expansile mass arising from cricoid cartilage	–	F-18 FDG PET/CT
				Lytic lesions at first and ninth ribs and T10 vertebra corpus.		Increased tracer uptake in subglottic larynx (SUVmax 2.9).
						Multiple skeletal increased tracer uptakes in the 1, 6, 9th ribs and T10 vertebra (SUVmax 3.6).
7	Plasmocytoma-multiple myeloma involvement	30	Supraglottic + glottic + subglottic	Transglottic mass, thyroid and cricoid cartilage destruction.	–	F-18 FDG PET/CT
				Multiple lytic skeletal lesions.		Multiple skeletal increased tracer uptakes (SUVmax 10.9).
8	Plasmocytoma-Multiple myeloma involvement	28	Subglottic	Lytic expansile mass arising from cricoid cartilage.	–	–
				Multiple lytic skeletal lesions.		
9	Adenocarcinoma metastasis	14	Glottic	Mass on the right vocal cord	–	–
				Bilateral level 2 metastatic lymphadenopathy.		
10	Paraganglioma	12	Supraglottic and left carotid bifurcation	Well defined, hypervascular mass at right preepiglottic space.	T1W isointense, T2W hyperintense, homogeneously enhancing mass lesions at right preepiglottic space and left carotid bifurcation	68Ga-DOTA-peptide PET/CT
				Hypervascular mass at left carotid bifurcation.		Right preepiglottic mass; Intense tracer uptake (SUV max: 35.8)
						Left carotid body mass; Intense tracer uptake (SUV max: 37.5)
						DSA:
						Right superior thyroid artery supplied laryngeal mass
						Left ascending pharyngeal artery supplied mass at left carotid bifurcation.
11	Chondroma	50	Supraglottic + glottic + subglottic+	Transglottic expansile mass arising from cricoid cartilage	T1W hypointense, T2W hyperintense, minimally heterogeneous enhancing mass	–
12	Laryngeal lipoma	22	Supraglottic + glottic	–	Supraglottic and glottic nonenhancing mass with typical fat signal characteristics: T1W hyperintense, entirely suppressed on fat-suppressed images.	–
13	Inflammatory myofibroblastic tumor	18	Glottic	Mass on the left vocal cord	–	–
14	Brown tumor of hyperparathyroidism	20	Subglottic	Expansile mass arising from cricoid cartilage.	–	–
				Maxillary and mandibular lytic expansile mass lesions		
				Parathyroid adenoma at left inferior gland location.		
15	Wegener's granulomatosis involvement	–	Diffuse soft tissue involvement	Diffuse edema and increased soft tissue thickness at laryngeal soft tissue.	–	–
				Lung CT: ground glass opacities, consolidation, focal bronchiectasis, nodules, bronchial and tracheal wall thickening.		
16	Tuberculous involvement	10	Supraglottic	Mass on left band ventricle, arytenoid cartilage sclerosis.	–	–
				Lung CT: right hilar lymphadenopathy, consolidations, ground glass opacities, bronchiectasis at left upper lob.		
17	Behçet's disease involvement	10	Supraglottic	Increased soft tissue thickness on left aryepiglottic fold and priform sinüs.	–	–
18	Vasculary malformation (low-flow)	47	Supraglottic + glottic	–	T1W hypointense, T2W hyperintense, enhancing mass right side of the supraglottic and glottic level of larynx and oropharyngeal, lingual, nasopharyngeal extensions.	–

CT, Computed Tomography; MRI, Magnetic Resonance Imaging; T1W, T1 weighted; T2W, T2 weighted; F-18 FDG PET/CT, Fluorine-18 Fluorodeoxyglucose Positron Emission Tomography/Computed Tomography; 68Ga-DOTA-peptide PET/CT, Gallium-68 DOTA-peptide Positron Emission Tomography/Computed Tomography; DSA, Digital Subtraction Angiography.

**Figure 4:**
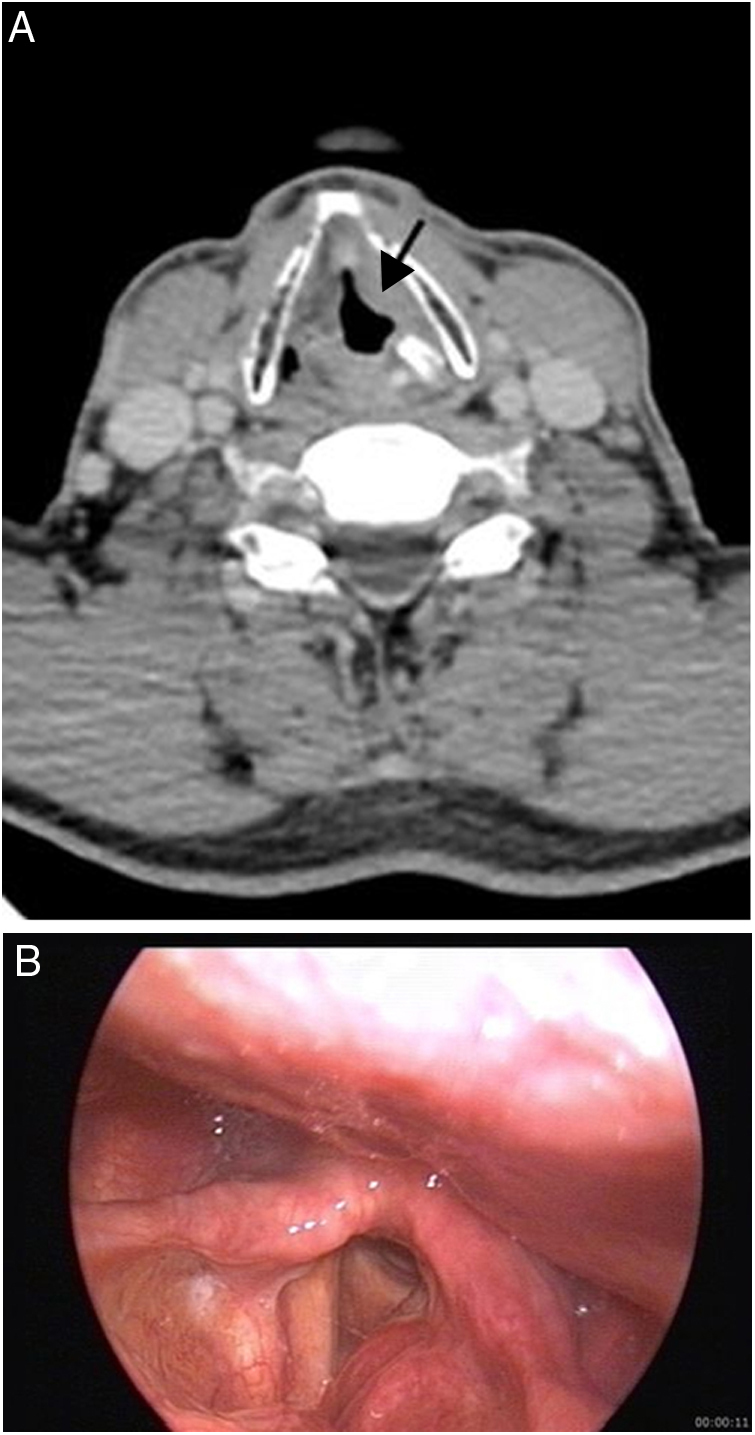
(A and B) 61 year-old man with pulmonary and laryngeal tuberculosis. Contrast enhanced CT image (A) shows a mass on the left band ventricle (arrow) and left arytenoid cartilage sclerosis. Endoscopic image (B) demonstrates a mass on the left band ventricle protruding on the left vocal cord.

**Figure 5:**
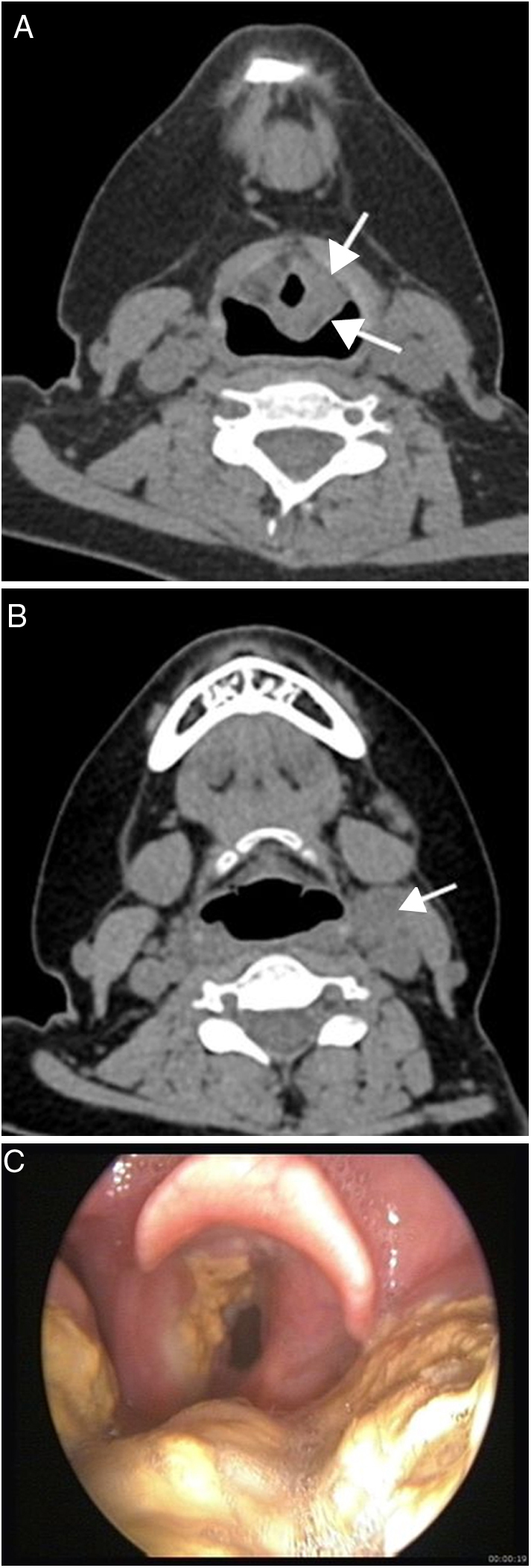
(A–C) 23 year-old woman with posttransplant lymphoproliferative disease, monomorphic, diffuse large B-cell type. Non-enhanced CT shows mass (arrows) (A) on left aryepiglottic fold and left cervical lymphadenopathy (arrow) (B). Endoscopic image (C) reveals irregular yellowish mass involving the left band ventricle, aryepiglottic fold and tongue base.

Imaging characteristics of laryngeal lesions are summarized in Table 2. Grade 2 chondrosarcoma was seen as hypodense mass with central necrosis and extralaryngeal extension on CT. Paraganglioma was seen as hyperdense on CT and homogeneous intense enhancing lesion on MRI due to the hypervascular nature of the lesion. Other benign and malignant lesions were seen as soft tissue density lesions on CT (Figure 4, Figure 5). Whereas WG and Behçet's disease involvements presented as soft tissue thickening, other lesions were seen as mass lesions. Chondroid matrix calcifications were detected in chondroma and chondrosarcoma-grade 1 on CT and these lesions had very high signal intensities on T2W images because of high water content of the hyaline cartilage. In the patient with vascular malformation, a typical very high signal intensity on T2W images was seen and the lesion showed diffuse extension from larynx to nasopharynx. Laryngeal lipoma showed typical MRI imaging characteristics including T1W hyperintensity, entirely suppressed on fat-suppressed images.

In addition, in the patient with paraganglioma, 68Ga-DOTA-peptide PET/CT was performed in order to confirm the diagnosis and to disclose any possible focus of paraganglioma on the other site. Both lesions showed intense tracer uptake (larynx – SUV max: 35.8, carotid body SUV max: 37.5) and no additional focus was identified. DSA demonstrated right superior thyroid artery supplied laryngeal mass and left ascending pharyngeal artery supplied mass at the left carotid bifurcation.

## Discussion

Non-SCC involvements of larynx are relatively rare conditions and treatment options for these diseases differ from SCC. In the present study, we evaluated clinical presentations, physical examination findings, imaging findings and treatment methods of patients with rare laryngeal diseases including 9 malignant neoplasms (2 chondrosarcoma, 1 neuroendocrine tumor-atipical carcinoid, 1 NK/T-cell lymphoma, 1 diffuse large B-cell lymphoma, 3 plasmocytoma-multiple myeloma involvement, 1 adenocarcinoma metastasis), 3 benign neoplasms (chondroma, paraganglioma, lipoma), 2 tumor-like lesions (Brown tumor and Inflammatory Myofibroblastic Tumor (IMFT), 3 inflammatory lesions (WG, Behçet's disease and tuberculosis involvements), and 1 vascular malformation.

There are a limited number of studies in the English literature of non-squamous carcinomas and uncommon diseases of the larynx. Cady et al.^6^ reported clinical findings and treatment methods of 31 non-epidermoid cancers of the larynx in 1968. Gadomski et al.^7^ published clinical findings and treatment methods of 19 non-epidermoid carcinomas of the larynx in 1986. Becker et al.^8^ reported radiologic–pathologic correlation of 40 non-squamous neoplasms of the larynx in 1998. Lin et al.^9^ reported staging and survival analysis of 140 non-squamous cell carcinomas of the larynx in 2008. Thompson and Gannon^10^ reported clinicopathologic findings of 111 chondrosarcomas of the larynx in 2002. Ebihara et al.^11^ reported 33 cases of carcinoid tumors of the larynx in 2007. Telugu et al.^12^ published clinicopathological study of 18 cases of inflammatory myofibroblastic tumors in 2017. These articles are mostly related to clinicopathologic findings and treatment methods of laryngeal diseases except Becker et al.’s study that includes radiologic and pathologic features of non-squamous neoplasms of the larynx. Most of the other reports are case series in the English literature.

A patient with a laryngeal tumor might have symptoms like dysphonia, dyspnea, dysphagia, pain, coughs, halitosis and swelling in the neck. These symptoms vary according to the topographical site involved in the larynx. While an isolated glottic tumor would disrupt voice quality, a subglottic mass would likely cause shortness of breath. The clinical evaluation of a patient with one of these symptoms must include a detailed examination of the larynx, either with indirect or direct laryngoscopy as well as a complete head and neck examination. In the case of a submucosal mass especially in the supra or subglottic regions, a salivary gland or a lymphoid tumor must be ruled out. A chondroma or a chondrosarcoma would present as a fixed, firm mass with a normal overlying mucosa. A plasmocytoma can be seen as a polypoid mass, gray-red to deep red in color.^7^ Physical examination findings of the patients in the current study varied according to the type of the lesions and the involved laryngeal site. Inflammatory masses were observed as ulcerated or irregular masses with edema or deformity of the laryngeal structures. The lesion of the patient with a vascular malformation was observed as a submucosal mass but purplish in color, which could be clearly distinguished from the normal mucosa and the final diagnosis was confirmed when the clinical findings were conjoined with MRI findings.

Chondrosarcoma is the most common sarcoma of the larynx and constitutes 1% of laryngeal neoplams. They occur most frequently in men, between 50 and 70 years.^8^ Chondrosarcomas arise from cricoid cartilage (75%), thyroid cartilage (17%), arytenoid cartilage (5%), and epiglottis and accessory cartilages (3%).^13^ Laryngeal chondrosarcoma presents as a smooth, lobulated, submucosal mass covered by normal mucosa. Coarse-stippled calcifications within the mass lesion are suggestive for chondroid tumors on CT images. Chondroid tumors have very high signal intensity on T2 weighted MR images due to low cellularity and high water content of hyaline cartilage. It is difficult to distinguish between chondroma and low grade chondrosarcoma based on the imaging findings. However, lymph node metastasis and local invasion findings may suggest chondrosarcoma. In our study, Grade 1 chondrosarcoma and chondroma originated from cricoid cartilage and had very high signal intensity on T2 weighted MR images. Coarse-stippled calcifications were seen in chondroma and Grade1 chondrosarcoma. Grade 2 chondrosarcoma was seen as a transglottic mass with central necrosis. Also, the presence of cricoid cartilage expansion, thyroid and cricoid cartilage destruction, extralaryngeal extension and lymph node metastasis were suggestive of chondrosarcoma in this case.

Two neuroendocrine neoplasms were present in our study; paraganglioma and atypical carcinoid tumor (Grade 2). Both were supraglottic and submucosal masses. Paraganglioma was seen as hypervascular, well defined mass in the preepiglottic space on CT and MRI and showed intense tracer uptake on 68Ga-DOTA-peptide PET/CT. Atypical carcinoid tumor was seen as a nonspecific soft tissue mass on the right aryepiglottic fold and interarytenoid region. Neuroendocrine neoplasms of the larynx are most common non-SCC neoplasms of the larynx and account for 1of all laryngeal neoplasms.^9^ Laryngeal neuroendocrine neoplasms are divided into 2 main categories: neural-benign (paraganglioma) and epithelial-malignant (typical carcinoid, atypical carcinoid, small-cell neuroendocrine carcinoma).^14^ It was reported that most of paragangliomas are seen as a supraglottic hypervasculary mass.^15^ Zhu et al.^16^ analyzed 14 laryngeal neuroendocrine carcinomas. They reported that these tumors present as a submucosal mass with mostly supraglottic location. Atypical carcinoids and especially small-cell neuroendocrine carcinomas may cause cervical lymphadenopathy and distant metastasis.^17^ There was no cervical lymphadenopathy in patients with atypical carcinoid tumor.

Primary Non-Hodgkin Lymphoma (NHL) of the head and neck mostly arises from the extranodal lymphatic system of Waldeyer's ring. However, extranodal extralymphatic sites involvement occurs in 25% of these tumors.^18^ The most common extranodal extralymphatic sites are sinonasal tract, salivary glands, thyroid and orbit. Primary laryngeal NHL is a rare condition and accounts for <1% of laryngeal tumors.^18^ B-cell phenotype is more common than T-cell (ratio of 6:1).^18^ Cervical lymphadenopathy may be seen in a quarter of the cases. It was reported that a large, submucosal, supraglottic, moderate enhanced mass with hypopharyngeal, oropharyngeal extension should suggest laryngeal NHL.^18^, ^19^ In the present study, laryngeal NHL (NK/T cell) was seen as supraglottic (epiglottis, aryepiglottic fold, band ventricle) centered mass with glottic and hypopharyngeal extension and ipsilateral level 2 metastatic lymphadenopathy.

The other lymphoma case was a posttransplant (kidney) monomorphic diffuse large B-cell lymphoma that was seen as nonspecific mass on left aryepiglottic fold, band ventricle with ipsilateral level 3 metastatic lymphadenopathy. Additional site involvements were detected during diagnosis such as lung, spleen, stomach, breast, hilar and mediastinal lymph nodes involvements. Posttransplant Lymphoproliferative Disease (PTLD) comprises a heterogeneous group of disease. According to World Health Organization (WHO) classification, PTLD is divided into three categories: early lesions, polymorphic PTLD, and monomorphic PTLD.^20^ In monomorphic PTLD, the most aggressive phase of disease, 80% of cases are of B-cell origin. The most common subtype is diffuse large B-cell lymphoma. Although fever and lymphadenopathy are most common presentations, extranodal involvement, central nervous system involvement may occur. Laryngeal involvement is rare condition and only a few cases have been reported.^21^ Specific imaging findings have not been described. But, after the solid organ transplantation, presence of upper airway obstruction symptoms should alert the clinician to possibility of PTLD.

In the present study, 3 of 9 malignant neoplasms were multiple myeloma involvements. A lytic expansile mass arising from cricoid cartilage was seen in two patients. In the third patient, a transglottic mass with thyroid and cricoid cartilage destruction was seen on CT images. In the literature, only a few cases of multiple myeloma involvement have been reported and the location of lesions were reported as aryepiglottic fold, band ventricle, vocal cord and subglottic region.^22^ Primary extramedullary plasmocytoma is a localized monoclonal plasma cell tumor without evidence of multiple myeloma at diagnosis. Laryngeal extramedullary plasmocytoma accounts for 0.04% to 0.19% of malignant laryngeal neoplasms.^23^ Distinguishing between these two types of plasma cell tumors is important because of their different treatment modalities.

Metastatic involvement of the larynx is a rare condition. The most common primary tumors metastasing to the larynx are malign melanoma and renal carcinoma.^24^ Breast, lung, prostate, stomach and colon metastases have also been reported.^8^, ^25^ Radiological findings of laryngeal metastasis are usually nonspecific. However, melanotic melanoma metastasis displays T1 hyperintensity and T2 intermediate to low signal intensity due to melanin content.^8^ Renal adenocarcinoma metastasis is hypervascular and shows strong enhancement. The most common sites of metastasis are the supraglottis and subglottis because of rich lymphatic and vascular supply. In the present study, laryngeal metastasis was localized on the glottis. Histopathologic features were similar with previously operated lung adenocarcinoma. There were also bilateral cervical metastatic lymph nodes.

One of the 2 tumor-like lesions of the present study was IMFT that was seen as nonspecific mass lesion on the left vocal cord. Concomitantly, squamous cell carcinoma was also present at the anterior commissure on histopathological examination. In the current WHO classification of tumors of soft tissue and bone, IMFTs are classified as intermediate grade tumors.^26^ Their behavior ranges from benign to malignant. Head and neck IMFTs account for 14%–18% of all extra-pulmonary IMFTs and the larynx is the most common site in the head and neck for IMFT.^27^ No specific imaging sign has been described for IMFTs in the literatüre. Vocal cords are the most common sites for laryngeal IMFT.^28^ SCC and IMFT coexistence is a very rare condition. As far as we know, this case is the second reported case of this coexistence. The other case was reported by Suzuki et al.^29^

Brown tumor of the larynx is a very rare condition. There are only 2 case reports in the literature with laryngeal Brown tumor, one of which is in the thyroid and the other is in the cricoid cartilage.^30^, ^31^ In the present study, Brown tumor was seen as well-defined, expansile, noncalcified, homogeneous enhanced lesion in cricoid cartilage with narrowing of the subglottic airway. Brown tumor is non-neoplastic proliferative disorder that occurs as a result of primary or secondary hyperparathyroidism. The lesion can arise in long or flat bones as a solitary or multiple lesions and most common sites are femur, tibia, pelvis, ribs and mandible. In our case, the etiology of Brown tumor was parathyroid adenoma and additional bone lesions were present.

Of the 3 inflammatory lesions, Behçet's disease and WG involvements presented as increased soft tissue thickness and edema on CT. Tuberculosis involvement was seen as a mass lesion on left band ventricle and arytenoid cartilage sclerosis. Laryngeal involvement of Behcet's disease is a very rare condition compared to laryngeal tuberculosis and laryngeal involvement of WG. Although Behcet's disease is defined as classical the triad of oral and genital ulcerations and inflammatory eye diseases, joints, vascular structures, nervous system, ear, nose and throat may also affected. There are only a few cases of laryngeal involvement of Behcet's disease in the English literature, including laryngeal ulcerations and stenosis.^32^ In our case, ulceration, increased soft tissue thickness and chronic-active inflammation were detected on clinical, imaging and histopathologic examination; the patient received medical treatment.

Airway involvement occurs in 15%–55% of patients with WG and this may be the only or the presenting finding of WG. Laryngeal and tracheal ulcers, subglottic stenosis may be seen in 25% and 16% of patients, respectively.^33^ Airway involvements may lead to severe obstruction, which can be fatal. Airway involvement may be focal or diffuse, circumferential or irregular. In our case with WG, diffuse tracheal and bronchial wall thickening, left bronchial stenosis, diffuse laryngeal edema, laryngeal soft tissue thickening were found. Additional lung involvement findings such as consolidations, ground glass opacities, focal bronchiectasis were seen on serial CT examinations.

The larynx is the second most common site for head and neck tuberculosis after the cervical lymph nodes.^34^ Laryngeal tuberculosis in cases of pulmonary tuberculosis has become very rare since the advent of antituberculosis chemotherapy and the incidence was reported as less than 1% of tuberculosis cases.^35^ Vocal cords are the most common region for laryngeal tuberculosis. Five different lesion types were reported: perichondritic, ulcerative, granulomatous, polypoid and nonspecific inflammatory.^36^ Left vocal cord and ventricle were affected in our case. Perichondritis was seen as arytenoid sclerosis on CT images. Granulomatous inflammation was described on histopathology and accompanying lung findings were present.

The cases of vascular malformation and lipoma were diagnosed based on the clinic and typical imaging characteristics of lesion in the present study. Vascular malformations have T2 hyperintensity and high-flow lesions show early strong enhancement after contrast administration. Endoscopic biopsy is not recommended in cases with vascular malformation due to the bleeding risk.

The main limitations of our study are its retrospective design and small number of patients.

## Conclusion

This unique study evaluates 18 didactic cases and points that diverse benign and malignant tumors of epithelial, mesodermal and neuroectodermal origin, tumor-like conditions and inflammatory diseases may affect the larynx. Imaging studies may provide clues for diagnosis of non-SCC laryngeal lesions. Although clinical and imaging findings are nonspecific in some of them, combined analyses of clinic, endoscopic, imaging findings and previous clinical history may contribute to precise final diagnosis and clinical management.

## Conflicts of interest

The authors declare no conflicts of interest.
